# Teaching in the clinical workplace: looking beyond the power of ‘the one’

**DOI:** 10.1007/s40037-015-0179-7

**Published:** 2015-05-20

**Authors:** Renée E. Stalmeijer

**Affiliations:** School of Health Professions Education, Maastricht University, PO Box 616, 6200 MD Maastricht, The Netherlands

The question ‘what is good clinical teaching?’ has intrigued medical education researchers for decades. We have tried to determine the characteristics of good clinical teachers, and have sought ever better ways to train and evaluate them. These research efforts have brought forward a myriad of publications that have resulted in faculty development programmes, quality improvement efforts and even frameworks for promotion and tenure.

Clinical teaching and all efforts to improve it are strongly influenced by the fact that they are situated within a workplace context in which patient-centred care is increasingly dependent on the collaboration of interprofessional health care teams. Add time pressure and budgetary constraints to the mix and one gets a perfect storm which we like to call the clinical learning environment. It is in this environment that medical students are socialized into their chosen profession.

Teaching and supervision are essential to the learning process of students in the clinical workplace because students need feedback and support to help structure their learning experiences and reflect on their competence development. What strikes me, however, is that our efforts to improve clinical teaching consistently focus on individual clinical teachers. Some results from more recent research indicate that it would perhaps be more naturalistic to approach clinical teaching as a team effort and not primarily as a solo endeavour [1, 2]. And moreover, some findings suggest that it could be worthwhile to move beyond our focus on physicians as the only teachers of medical students, and to embrace the teaching potential of other health care professionals who operate in the clinical learning environment [3, 4].

Looking beyond the one-on-one relationship between a student and a teacher and widening our scope to the level of team or community of practice, there is a larger movement visible within the medical education domain. Alan Bleakley, in his 2006 paper on ‘broadening conceptions of learning in medical education: the message from teamworking’, advocated a stronger focus on socio-cultural models of learning over ‘individualistic learning models’ to ‘explain how learning occurs in dynamic, complex and unstable systems such as fluid clinical teams’ [5].

So let us further explore the *clinical teaching team* concept: how could we define it and what would its potential research implications be?

Patient care is a team effort. As students (be they medical students or residents) learn by participating in this team effort, they are exposed to various (allied) health care professionals. Where some of these professionals might be more focused on the students’ clinical reasoning and medical knowledge, other professionals will focus on the students’ communication and collaboration skills. For example, the role of nurses in facilitating students’ understanding of workflow and ‘how things work around here’ seems to be common knowledge and even taken for granted. But what does this facilitation actually entail? The focus on the individual physician as the clinical teacher creates a blind spot with regard to the potential of the interprofessional health care team at large to function as ‘teacher’, and thus to the distributed nature and multiplicity of teaching within the workplace. The team perspective highlights the notion that all these health care professionals potentially have something to offer and can complement one another’s individual competencies to train medical students and residents.

The communities of clinical practice (CoCP) perspective offers a potential theoretical research avenue for exploring the clinical teaching team concept [6]. CoCP opens up the boundaries of the original communities of practice concept to move from a mono-professional entity to a multi-professional entity. The unifying element in both concepts is the activities of the community in pursuit of a shared enterprise. In a health care setting this shared enterprise can be defined as patient care. Understanding the interplay and dynamic within the community of clinical practice might help create a richer picture of the clinical learning environment and the potential ‘teachers’ within this environment.

From a more pragmatic point of view one could use the CanMEDS roles, or any other competency framework, as a lens to understand how residents learn to communicate and collaborate interprofessionally, and to examine the role that allied health professionals play in this process. Or how the community of clinical practice interplay teaches residents to become leaders or more effective health advocates?

Although the role of allied health care professionals in the learning process of students is readily recognized, professional boundaries and hierarchy within the clinical workplace will have likely created a barrier to explore the potential of the clinical teaching team. The physician has long been seen as ‘the one’: the one who is solely responsible for teaching medical students to become medical doctors. I feel that it might be worthwhile to try and look beyond professionals boundaries and include the allied health professionals in our understanding of who might fulfill the role of clinical teachers. Looking beyond the power of ‘the one’ might just help medical students to navigate their perfect storm.
